# Serotonin Syndrome Without Overdose: Polypharmacy-Induced Toxicity in a Medically Stable Young Adult

**DOI:** 10.7759/cureus.86587

**Published:** 2025-06-23

**Authors:** Andy Burk, Abbas Merchant, Haashim Rahman, Aaryan Patel, Ishan Deshmukh, Rida Merchant, Aaron Yam, Amy Yu, Chris Cai, Abbas Hassam, David Le, Constantino G Lambroussis

**Affiliations:** 1 Internal Medicine, Albany Medical College, Albany, USA; 2 Anesthesiology, Lake Erie College of Osteopathic Medicine, Erie, USA; 3 Physical Medicine and Rehabilitation, Lake Erie College of Osteopathic Medicine, Erie, USA; 4 Vascular Surgery, Lake Erie College of Osteopathic Medicine, Elmira, USA; 5 Anesthesiology, Albany Medical College, Albany, USA; 6 Anesthesiology and Critical Care, University of California San Diego, San Diego, USA; 7 Anesthesiology and Critical Care, Albany Medical College, Albany, USA; 8 Anesthesiology, Albany College of Medicine, Albany, USA; 9 Osteopathic Medicine/Family Medicine, Lake Erie College of Osteopathic Medicine, Elmira, USA

**Keywords:** altered mental state, antipsychotic polypharmacy (apps), bupropion, critical care psychiatry, escitalopram, naltrexone, serotonin syndrome diagnosis, serotonin syndrome (ss), toxic encephalopathy

## Abstract

Serotonin syndrome is a potentially life-threatening condition caused by excessive serotonin activity in the central and peripheral nervous systems that can lead to symptoms such as altered mental status, autonomic instability (e.g., hyperthermia and hypertensive tachycardia), and neuromuscular abnormalities (e.g., clonus and tremor). We present the case of a 29-year-old male healthcare worker with a history of major depressive disorder (MDD), temporal lobe epilepsy, and suspected obsessive-compulsive disorder (OCD) who presented with sudden-onset agitation, confusion, and abnormal limb movements after his third consecutive 12-hour overnight shift. He had been on stable outpatient doses of escitalopram 20 mg and bupropion XL 150 mg daily for over six months, with naltrexone 50 mg added three months earlier for off-label psychiatric use. In the emergency department, he was treated for presumed seizure or psychosis with multiple sedatives and antipsychotics and subsequently intubated. EEG showed no epileptiform activity, and lumbar puncture was unremarkable. Neurologic findings, including hyperreflexia, clonus, and rigidity, along with rapid clinical improvement following cyproheptadine and benzodiazepines, supported a diagnosis of serotonin syndrome. The patient denied overdose or substance use, and toxicology review concluded that polypharmacy with serotonergic and modulating agents was the likely trigger. We present a young adult who developed serotonin syndrome without overdose, likely due to the cumulative serotonergic effect of multiple prescribed medications at therapeutic doses. This case emphasizes the importance of clinical vigilance in patients with neuropsychiatric comorbidities on centrally acting agents, even in the absence of recent medication changes. It illustrates that even therapeutic combinations of serotonergic and modulating agents can precipitate life-threatening toxicity.

## Introduction

Serotonin syndrome is a potentially life-threatening condition caused by excessive serotonergic activity in the central nervous system, most often triggered by medication overdose, polypharmacy, or drug interactions involving selective serotonin reuptake inhibitors (SSRIs), serotonin-norepinephrine reuptake inhibitors (SNRIs), monoamine oxidase inhibitors (MAOIs), or other serotonergic agents [1-3]. Serotonin syndrome affects approximately 0.01-0.02% of patients treated with serotonergic medications. Among patients who present with an SSRI overdose, approximately 14-16% have concomitant serotonin syndrome, though its incidence is likely underreported due to nonspecific clinical features and a lack of awareness among healthcare providers [1,4].

The pathophysiology of serotonin syndrome involves an overabundance of serotonin, leading to the excessive stimulation of 5-HT1A and 5-HT2A receptors within the central nervous system [5]. Serotonin syndrome is typically a clinical diagnosis based on the patient’s presentation and pertinent history, particularly the pharmacological history of recent use of serotonergic medications. To aid in decision-making, specific guidelines such as the Hunter Serotonin Toxicity Criteria, which emphasize altered mental status, autonomic instability, and neuromuscular hyperactivity (including inducible clonus and hyperreflexia), can be used [6]. Furthermore, lab values like elevated creatine kinase, leukocytosis, and transaminitis may point to severe toxicity and end-organ damage. When considering other potential differential diagnoses, such as neuroleptic malignant syndrome, malignant hyperthermia, anticholinergic toxicity, and other infectious or structural etiologies, these must also be ruled out by physical findings and patient history, imaging, and analysis of cerebrospinal fluid. The treatment of serotonin syndrome necessitates the prompt cessation of all potentially contributing medications, coupled with supportive measures such as intravenous fluid administration. Benzodiazepines are typically the first-line treatment for sedation, with cyproheptadine or other serotonergic antagonists added in more severe or treatment-resistant cases [5].

Although serotonin syndrome typically arises in the context of dose escalation or polypharmacy involving serotonergic drugs, it can rarely occur at therapeutic dosing in patients with no recent changes to their medication regimens. Reports of severe serotonin syndrome in patients on stable therapeutic regimens are scarce, particularly in medically stable young adults with chronic psychiatric and neurologic comorbidities [7-11]

This case adds to the literature by illustrating how polypharmacy alone, even at standard therapeutic doses, can induce severe toxicity. We present a case of serotonin syndrome in a young adult male on long-standing therapeutic doses of escitalopram, bupropion, and naltrexone, who developed life-threatening serotonin toxicity without overdose or recent dose adjustment. His presentation was initially misinterpreted as a seizure, psychotic break, or infectious encephalopathy before rapid resolution with cyproheptadine confirmed the diagnosis.

## Case presentation

A 29-year-old male with a history of major depressive disorder (MDD), temporal lobe epilepsy, and suspected obsessive-compulsive disorder (OCD) presented from an outside hospital (OSH) with acute altered mental status, psychomotor agitation, and abnormal limb movements. He had reportedly been working his third consecutive 12-hour overnight shift as a nurse when he developed sudden-onset anxiety, crying spells, and confusion. Per chart review, he became increasingly disorganized and emotionally labile, expressing delusional fears of dying and demanding resuscitation. He exhibited generalized tremor, hyperventilation, and bilateral limb shaking.

At the emergency department of the OSH, he was administered multiple sedatives, including hydroxyzine, haloperidol, several doses of lorazepam, and diphenhydramine for a suspected dystonic reaction. He subsequently became minimally responsive and required intubation for airway protection. Routine labs were notable for a positive urine drug screen for benzodiazepines and amphetamines, which were a false positive, as corroborated by a confirmatory follow-up urine drug screen. A computed tomography (CT) scan of the head and lumbar puncture were performed and indicated no pathology. He was transferred to our institution for higher-level care.

**Figure 1:**
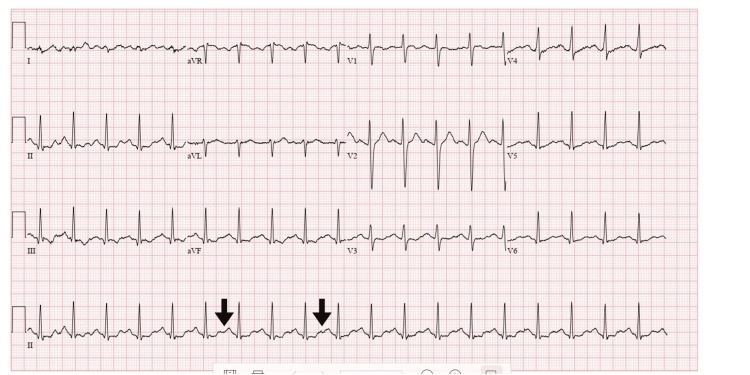
ECG performed on admission An ECG that was performed at the initial admission showed sinus tachycardia with nonspecific ST/T wave changes, as noted by the black arrows, and a QTc of 464 ms. No signs of ischemia, arrhythmia, or other cardiac pathology were present to explain the patient’s altered mental status. The features of autonomic instability were consistent with serotonin syndrome.

Upon arrival and admission, an ECG was performed and showed sinus tachycardia with nonspecific ST/T wave changes (Figure 1) as he remained sedated and intubated on continuous benzodiazepine and propofol infusions. Neurologic examination revealed inducible clonus in the lower extremities and diffuse hyperreflexia. EEG showed disorganized background activity and stimulus-induced clonus without epileptiform discharges.

The patient’s serum creatinine, lactate, creatine kinase, and other electrolytes were within normal limits. Collateral history from the patient’s mother revealed no substance use, suicidal ideation, or medication overdose. The patient had been on stable outpatient doses of escitalopram 20 mg daily and bupropion XL 150 mg daily for several months. Naltrexone 50 mg daily had been added approximately three months earlier by his psychiatric nurse practitioner for off-label treatment of compulsive behaviors. He had a history of seizure disorder managed with oxcarbazepine but was inconsistently adherent due to insurance-related challenges. His mother denied knowledge of him taking more than prescribed of any of his medications and reported no access to illicit substances at home.

Utilizing a bedside Hunter diagnostic criteria score based on the patient’s presentation and symptoms of clonus with agitation and diaphoresis, hypertonia/hyperreflexia, the decision to initiate treatment with cyproheptadine was made. Over the course of hospitalization, the patient showed rapid clinical improvement following initiation of cyproheptadine and down-titration of sedation. He was extubated on hospital day 2 and regained full orientation, reporting amnesia for the events prior to and during hospitalization. He denied hallucinations or delusional thought content and exhibited appropriate mood and affect. The patient also confirmed adherence to his prescribed doses of escitalopram, bupropion, and naltrexone. Psychiatry assessed him as psychiatrically stable, with no indication of overdose, substance use, or acute psychiatric decompensation.

**Figure 2:**
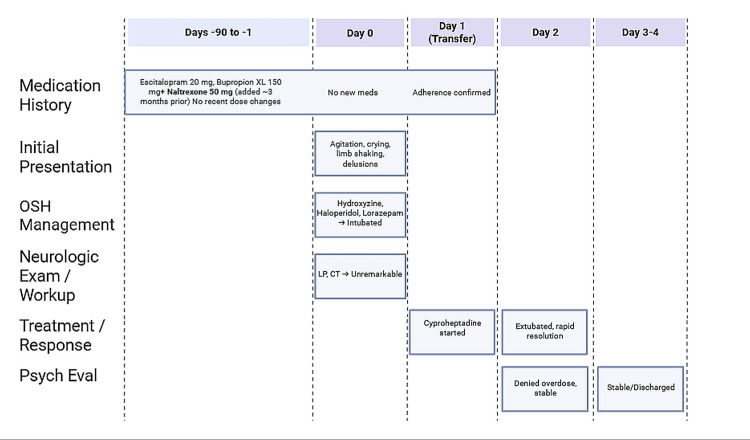
Timeline of clinical course Timeline of clinical course in a 29-year-old male with serotonin syndrome on long-standing serotonergic and modulating agents (escitalopram, bupropion, and naltrexone). Note the absence of overdose or recent dose change, with rapid improvement following cyproheptadine initiation. OSH, outside hospital; LP, lumbar puncture; CT, computed tomography

Due to the initial presentation of dramatic neuropsychiatric symptoms of agitation, incoherent speech, delusions, and limb shaking, prompt concern for seizure, psychosis, or toxic/metabolic encephalopathy was indicated. Notably, the patient’s abrupt onset of symptoms began after a period of sleep deprivation and emotional stress without any changes to his medication regimen. The resolution of symptoms following cyproheptadine and supportive care strengthens the diagnostic evidence for serotonin syndrome, emphasizing that even patients with stable regimens may develop toxicity when multiple serotonergic and serotonergic-modulating agents are involved. A timeline of the patient’s clinical course can be observed in Figure 2. This case demonstrates the importance of recognizing serotonin syndrome across varied presentations.

## Discussion

Serotonin syndrome is a potentially life-threatening condition resulting from excess serotonergic activity in the central nervous system. It is most commonly associated with medication overdose, dose escalation, or drug interactions involving SSRIs, SNRIs, MAOIs, and certain opioids or recreational drugs [1]. Symptoms typically emerge within 24 hours of a serotonergic trigger and follow a triad of altered mental status, neuromuscular hyperactivity (e.g., clonus and hyperreflexia), and autonomic instability (e.g., fever and tachycardia) [6].

This case illustrates an atypical manifestation of serotonin syndrome occurring in a medically stable young adult on long-standing outpatient regimens. The patient had been taking therapeutic doses of escitalopram and bupropion for over six months, with naltrexone added three months prior. All medications were reportedly taken as prescribed, and there was no evidence of overdose or illicit drug use. Despite the absence of classic precipitants, he developed acute-onset altered mental status, agitation, psychotic-like behavior, tremulousness, and inducible clonus, all of which resolved rapidly with cyproheptadine and supportive care.

What makes this case particularly noteworthy is the emergence of serotonin syndrome without any identifiable trigger in a patient on stable therapy. Escitalopram is a well-established serotonergic agent, but bupropion is not traditionally classified as such. However, bupropion may inhibit cytochrome P450 2D6 (CYP2D6), thereby increasing plasma concentrations of SSRIs [12-14]. Naltrexone, an opioid receptor antagonist commonly used for alcohol and opioid dependence, is not traditionally associated with serotonergic activity. However, there is emerging interest in its potential to influence neurotransmitter systems indirectly. In patients taking SSRIs, the concern arises that naltrexone might contribute to serotonin syndrome through complex pharmacodynamic interactions. While naltrexone does not directly increase serotonin levels, it may alter the balance of endogenous opioids and dopamine, potentially amplifying serotonergic transmission under certain conditions. Additionally, some studies suggest that opioid antagonism could modulate serotonergic pathways in the brainstem and hypothalamus. Though rare, case reports have documented serotonin syndrome in patients concurrently using naltrexone and other neuromodulation agents, suggesting a possible but poorly understood synergistic effect [9,15]. 

The patient also experienced a significant lack of sleep prior to the onset of his symptoms, although no direct link between sleep loss and serotonin syndrome has been proven, several indirect causes of increased serotonergic activity linked to poor sleep may have contributed to the presentation of serotonin syndrome seen in this patient. Studies have shown that in individuals with chronically poor sleep, serotonin turnover is increased with a concomitant modulation in 5‑HT₂A receptors, which may lead to overall increased serotonin available that may be implicated in predisposing to serotonin syndrome [16]. Together, this combination may have cumulatively lowered the threshold for toxicity, especially in the context of physiologic stress, in particular the lack of sleep in the patient, or metabolic variability.

The differential diagnosis included seizure, infectious encephalitis, medication-induced dystonia, and neuroleptic malignant syndrome, all of which were ruled out with a normal EEG, unremarkable cerebrospinal fluid, lack of rigidity, no hyperthermia, and no recent exposure to neuroleptics or anesthetics. The triad of clonus, hyperreflexia, and altered mental status, combined with rapid response to cyproheptadine, strongly supported the diagnosis of serotonin syndrome based on the Hunter Serotonin Toxicity Criteria [6]. Of note, the patient never developed hyperthermia, a symptom often associated with severe serotonin syndrome. However, up to one-third of serotonin syndrome cases may lack fever, as noted in the existing literature [13], making clinical suspicion critical even in normothermic presentations.

## Conclusions

This case shows that serotonin syndrome can occur even in the absence of overdose or recent medication changes, purely from the cumulative effect of multiple serotonergic and modulating agents taken at therapeutic doses. Atypical presentations, especially in young, psychiatrically stable patients, may mimic seizure or psychiatric decompensation, delaying diagnosis. Prompt recognition, withdrawal of serotonergic agents, and administration of cyproheptadine led to rapid resolution and full recovery, as was seen in our patient presentation. Clinicians should maintain a high index of suspicion for serotonin syndrome in patients with neuropsychiatric comorbidities on polypharmacy and routinely review serotonergic burden to prevent life-threatening toxicity. Furthermore, patient education on the risks of polypharmacy and the early signs of serotonin syndrome while taking psychiatric medications is highly important in the prevention of serotonin syndrome. Early recognition of serotonin syndrome and withdrawal of serotonergic medications can lead to rapid improvement and avoid further complications.
